# Nurses’ Recognition and Care of Thirst in Perioperative Patients in Japan

**DOI:** 10.7759/cureus.76624

**Published:** 2024-12-30

**Authors:** Mariko Hanashiro, Mayu Fukuda, Tomoko Akase

**Affiliations:** 1 Department of Biological Science and Nursing, Yokohama City University Graduate School of Medicine, Yokohama, JPN; 2 Department of Advanced Clinical Specialist Center, Kameda Medical Center, Kamogawa, JPN

**Keywords:** nursing research, post-anesthesia care unit, postoperative thirst, quality-of-care, recognition

## Abstract

Purpose: Postoperative thirst is common and distressing to patients, as is pain and nausea. The causes of postoperative thirst are complex and include factors like preoperative fasting, perioperative fluid loss, and certain anesthesia medications. Effective care for postoperative thirst has been shown in post-anesthesia care units (PACUs), but many Japanese hospitals lack PACUs or do not address thirst in their PACUs. Therefore, cooperation between the operating room and ward nurses is crucial for providing proper care for postoperative thirst. The purpose of this study was to clarify the actual situation of ward nurses' and operating room nurses' recognition and care of thirst in postoperative patients.

Methods: The study was a cross-sectional survey conducted using a self-administered questionnaire based on previous research. Study participants were nurses working in surgical wards and operating rooms of two university-affiliated hospitals. The survey items included (1) participants’ characteristics, (2) recognition of thirst in perioperative patients, and (3) actual care provided to perioperative patients for thirst. Data were collected between September and October 2022 and subjected to descriptive and bivariate analysis.

Findings: A total of 298 ward nurses and 43 operating room nurses were included in the study. Among the observation items, thirst was observed least frequently. Both ward nurses and operating room nurses recognized patient thirst based on complaints rather than physical observations. Sharing of information about patient thirst differed between ward nurses and operating room nurses. None of the participants used scales or scores to evaluate thirst. The most common postoperative care in the ward was “They were asked to do a mouthwash with water,” while in the operating room, it was “Placed a moistened gauze against the mouth.” The reasons for selecting a particular type of care were primarily based on “Because of the patient's wishes” and “I think it is effective,” while the reasons for not providing care included “Because there is an instruction not to drink water,” “Due to the possibility of aspiration,” “I don't have the knowledge and don't know how,” and “I'm busy with other work.”

Conclusions: Regarding the recognition of thirst, both ward nurses and operating room nurses recognized patient thirst most often when the patient directly reported feeling thirsty. They relied more on patients’ verbal complaints than physical observations to recognize thirst. Both surgical ward nurses and operating room nurses provided care based on their experience.

## Introduction

Between 75.0% and 89.6% of surgical patients experience thirst within 24 hours after surgery [1,2], making it a common complaint, along with pain, nausea, and vomiting during the perioperative period. The causes of postoperative thirst vary but may include age, sex, hormonal changes, anxiety, and the use of anesthesia and intraoperative drugs [3]. Thus, postoperative thirst cannot be easily improved by correcting circulating blood volume with fluid replacement alone. In addition, perioperative oral cavity-related complications include the risk of infection due to postoperative pneumonia, oropharyngeal wound infection, and periodontitis [4], and thirst and dry mouth may contribute to these complications.

Preoperative fasting for at least eight hours and abstaining from drinking water for two hours before the induction of anesthesia are strongly recommended by the American Society of Anesthesiologists to prevent vomiting and aspiration during surgery. However, many medical institutions enforce prolonged fasting, lasting an average of 12-16 hours [5], resulting in preoperative thirst in about 43%-69% of patients [6,7]. Perioperative thirst lasts for a long period of time from the preoperative stage to the postoperative stage; therefore, patients can experience stress due to the inability to drink water [2,8-10].

Overseas, it has been proven that there is a relationship between thirst and discomfort in patients after surgery [11], and methods for evaluating discomfort due to thirst have been clarified [12]. Care to improve perioperative thirst is being considered both before and after surgery. In preoperative care, chewing menthol gum has been demonstrated to improve perioperative thirst [13]. In postoperative care, post-anesthesia care unit (PACU) care has been shown to improve thirst without adverse events [14,15]. The ability of patients and healthcare providers to recognize thirst has also been clarified. Postoperative patients perceive thirst as distressing, and even if they report it to their healthcare providers, they may not receive adequate care [10]. However, healthcare providers tend to have a low interest in thirst, undervalue its importance, or do not give it much attention [9,13].

In Japan, previous studies on improving thirst during the perioperative period have mostly focused on improving care on the ward in specific departments, such as orthopedics, or specific surgeries, such as spinal surgery or laparoscopic cholecystectomy [16-18]. However, a perioperative patient satisfaction survey conducted in 2020 also found that postoperative thirst was the second most uncomfortable factor after pain, regardless of the type of surgery [19], indicating that patient discomfort has not been adequately addressed. Care that has been shown to be effective in previous studies is not used in daily clinical practice, and furthermore, it is also unclear how nurses who provide care recognize a patient's thirst. PACU is a system that provides immediate observation and nursing care after surgery and is considered standard and essential in the United States [20] and many European Union [21]. However, only 16.1% of hospitals in Japan have a PACU, and in many facilities, patients are generally transferred directly to the general ward after awakening on the operating table [22]. Therefore, collaboration between ward nurses and operating room nurses is important to provide nursing care based on the effects of surgery and anesthesia.

Recognizing patient discomfort due to thirst and promptly improving it is essential. Therefore, nurses should recognize thirst to initiate care. Knowing whether ward nurses and operating room nurses recognize patient thirst is necessary to improve thirst itself. Therefore, in this study, we aimed to clarify the actual status of perioperative patient thirst and the provision of nursing care for thirst, as recognized by ward nurses and operating room nurses in Japan.

## Materials and methods

Definitions of terms

In this study, “thirst” was defined according to Greenleaf’s definition [23] as “a sensation of dryness in the mouth and throat that is associated with a desire for liquids.” It should be noted that “dry mouth” is sometimes used as a translation for “口渇” in Japanese, but for the purpose of this study, we used the term “thirsty” to maintain consistency with the definition provided by Greenleaf [23].

Study design

This is a cross-sectional survey study using a self-administered questionnaire. The Strengthening the Reporting of Observational Studies in Epidemiology (STROBE) was chosen as a checklist for this study.

Study participants

Using the convenience sampling method, nurses working in surgical wards and operating rooms at two university-affiliated hospitals were selected as the study participants. Nursing managers and first-year nurses who were less likely to actually provide patient care were excluded from the study. Participants with incomplete or missing responses were also excluded.

Survey period

The survey period was from September 2022 to October 2022.

Survey procedure

The research purpose, method, and outline were explained to the study participants in writing, and only those participants who provided consent completed the questionnaire. The deadline for the responses was two weeks.

Survey content

The necessary survey items were created by reference to previous studies [16,24,25]. The question items were discussed among the researchers. After receiving supervision from experienced nurses in perioperative nursing and the head nurse of the operating room, the question items were modified. The questionnaire was pre-tested on 15 nurses with over two years of experience in surgical or operating room wards, and the questions were confirmed to have no problems in terms of the content or answers (see Appendix). The following items were assessed: (1) Background of the participants (gender, years of nursing experience, last school institution, qualifications other than nurses, and general anesthesia experience). (2) Questions regarding the observation items in the perioperative period, the factors causing thirst, and the evaluation of thirst. (3) Recognition and current care practices for preoperative thirst. (4) Recognition and current care practices for intraoperative thirst (non-intubated or intubated). (5) Recognition and current care practices for postoperative (after extubation) thirst.

Question item (4) was answered only by operating room nurses. For questions (3)-(5), to capture the actual care provided for thirst, the question items on the care provided and the frequency of care were answered based on the respondents' memory. In accordance with previous studies, the researchers discussed and asked about the care provided within the past month. For the observation items, recognition of thirst, and information sharing, a four-point Likert scale (4: Always (observe/recognize/share); 3: Sometimes (observe/recognize/share); 2: Rarely (observe/recognize/share); 1: Never (observe/recognize/share)) was used to obtain the responses. In this study, the objective was for nurses to objectively recognize patients’ subjective feeling of thirst as a third party.

Analytical methods

Quantitative data were calculated with means and standard deviations, and categorical variables were summarized using frequencies and percentages.

For survey items (1) and (2), simple tabulation was conducted and descriptive statistics were calculated. For survey items (3), (4), (5), and (6), in addition to simple tabulations, Likert scale 4 and 3 were analyzed as the (observe/recognize/share) group, and 2 and 1 as the (do not observe/recognize/share) group. For survey items (3), (4), (5), and (6), regarding the question on providing care for patients’ thirst, those who answered “provided care” were classified as the “care provided group,” and those who answered “did not provide care” were classified as the “no care provided group,” and simple tabulation was conducted. For the “care provided group,” questions were asked about the “type of care provided,” “frequency,” and “reasons for choosing care.” For the “no care provided group,” a question was asked about the “reasons for not providing care.” A “free description” item was provided for each question, a simple tabulation was conducted, and descriptive statistics were calculated.

Master charts were created using Microsoft Excel (Microsoft Corporation, Redmond, USA). Data were analyzed using Statistical Package for Social Science (SPSS) version 28 (IBM SPSS Software, IBM Corporation, Chicago, USA). 

Ethical considerations

This study was conducted with the approval of the Yokohama City University Research Ethics Committee for Life Sciences and Medicine involving human subjects (Approval No. F220900001).

## Results

Questionnaires were distributed to 507 ward nurses and 110 operating room nurses at the two facilities who cooperated in the survey. A total of 341 people, 298 surgical ward nurses and 43 operating room nurses, who responded validly, were included in the analysis.

Background of the participants 

The participants’ characteristics are presented in Table 1. Of these, 298 (87.4%) were surgical ward nurses and 43 (12.6%) were operating room nurses. The final education level of all analyzed subjects was vocational schools for 111 (102 ward nurses, nine operating room nurses) participants, junior colleges for 22 (19 ward nurses, three operating room nurses), universities for 199 (168 ward nurses, 31 operating room nurses), and graduate schools for eight (eight ward nurses). University graduates were the most common for both surgical ward nurses and operating room nurses. The number of years of nursing experience for all nurses was 12.1 ± 10.4 years. The number of years of experience for ward nurses was 12.6 ± 10.9 years, and for operating room nurses was 10.4 ± 8.2 years.

**Table 1:** Sample characteristics. The data are presented as n (%) Nursing experience in years (mean ± SD)

	Surgical ward nurse	Operating room nurse
	(n=298)	(n=43)
Sex		
Male	17 (5.7)	0
Female	279 (93.6)	43 (100.0)
Last school institution	0	0
High school	0	0
Vocational school	102 (34.2)	9 (20.9)
Junior college	19 (6.4)	3 (7.0)
University	168 (56.4)	31 (72.1)
Graduate school	8 (2.7)	0
Years of experience as a nurse	12.6 ± 10.9	10.4 ± 8.3
Qualifications other than nurses		
None	264 (88.6)	30 (69.8)
Certified nurse	9 (3.0)	1 (2.3)
Certified nurse specialist	1 (0.3)	0
Nurses involved in specific acts (including during training)	3 (1.0)	0
Perioperative management team certified nurse	1 (0.3)	7 (16.3)
Certified respiratory therapist	5 (1.7)	2 (4.7)
Others	14 (4.7)	4 (9.3)
Presence or absence of general anesthesia experience		
Experienced	70 (23.5)	12 (27.9)
No experience	228 (76.5)	31 (72.1)

Nurses’ recognition of patient thirst during the perioperative period

Postoperative Observation Items by Nurses in Routine Practice

**Figure 1:**
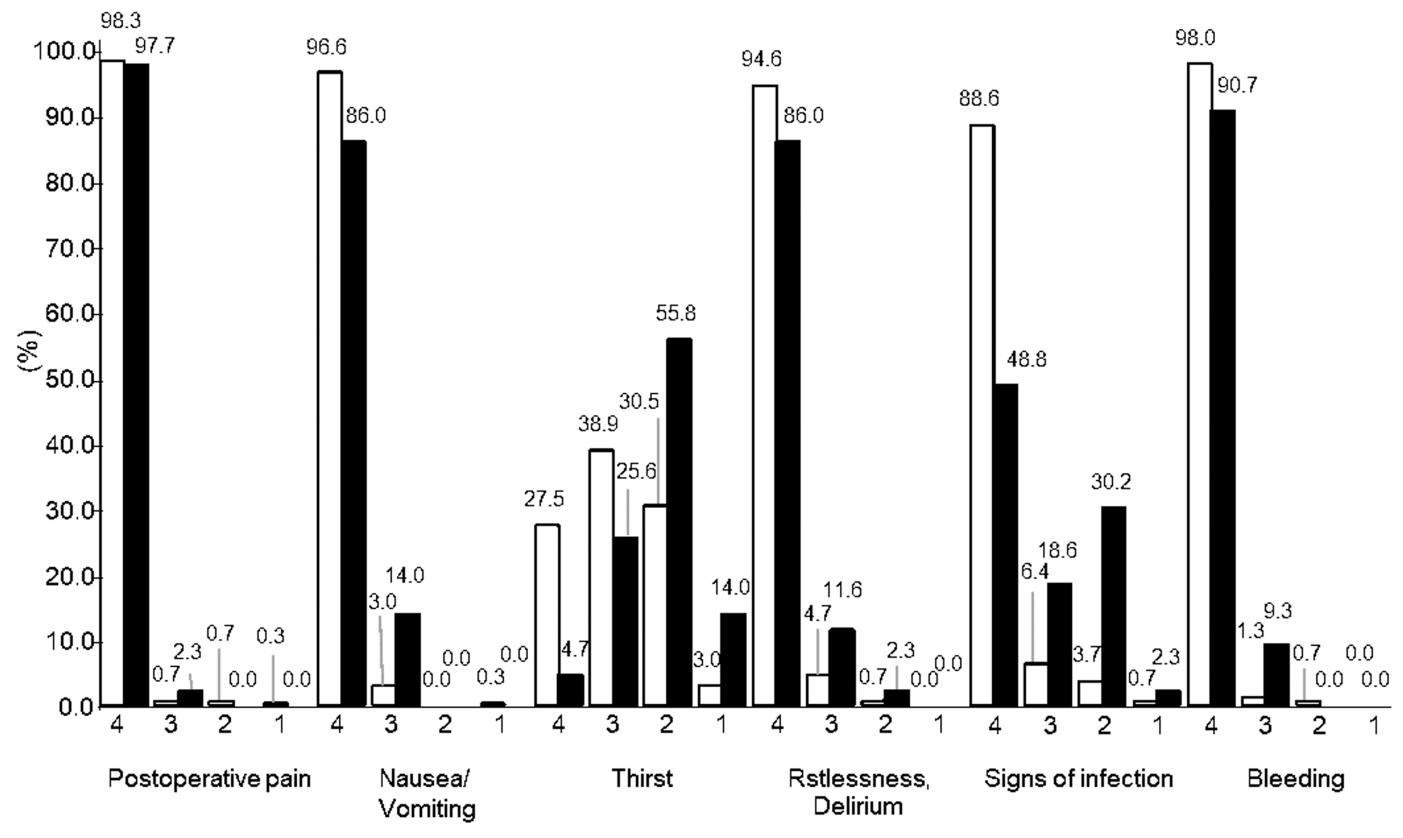
Postoperative observation items in routine practice. Surgical ward nurses (n=298), operating room nurses (n=43). 4: Always observe, 3: Sometimes observe, 2: Rarely observe, 1: Never observe. Light bars: Surgical ward nurses, Black bars: Operating room nurses.

Figure 1 shows the percentage of postoperative observation items observed by ward nurses and operating room nurses in daily clinical practice. The white bar graph shows the percentage of postoperative observation items observed by ward nurses, and the black bar graph shows the percentage of postoperative observation items observed by operating room nurses. Both surgical ward nurses and operating room nurses answered “always observe” for over 85% of the postoperative observation items, such as “postoperative pain,” “nausea and vomiting,” “restlessness and confusion,” and “bleeding.” “Thirst” had a lower percentage of “always observed” responses than the other observation items for both ward nurses and operating room nurses. The proportion of ward nurses who answered “observe sometimes” was the highest (38.9% (116 nurses)), while the proportion of operating room nurses who answered “do not observe much” was the highest (55.8% (24 nurses)).

Timing of Nurses’ Recognition of Patient Thirst During the Perioperative Period

**Figure 2:**
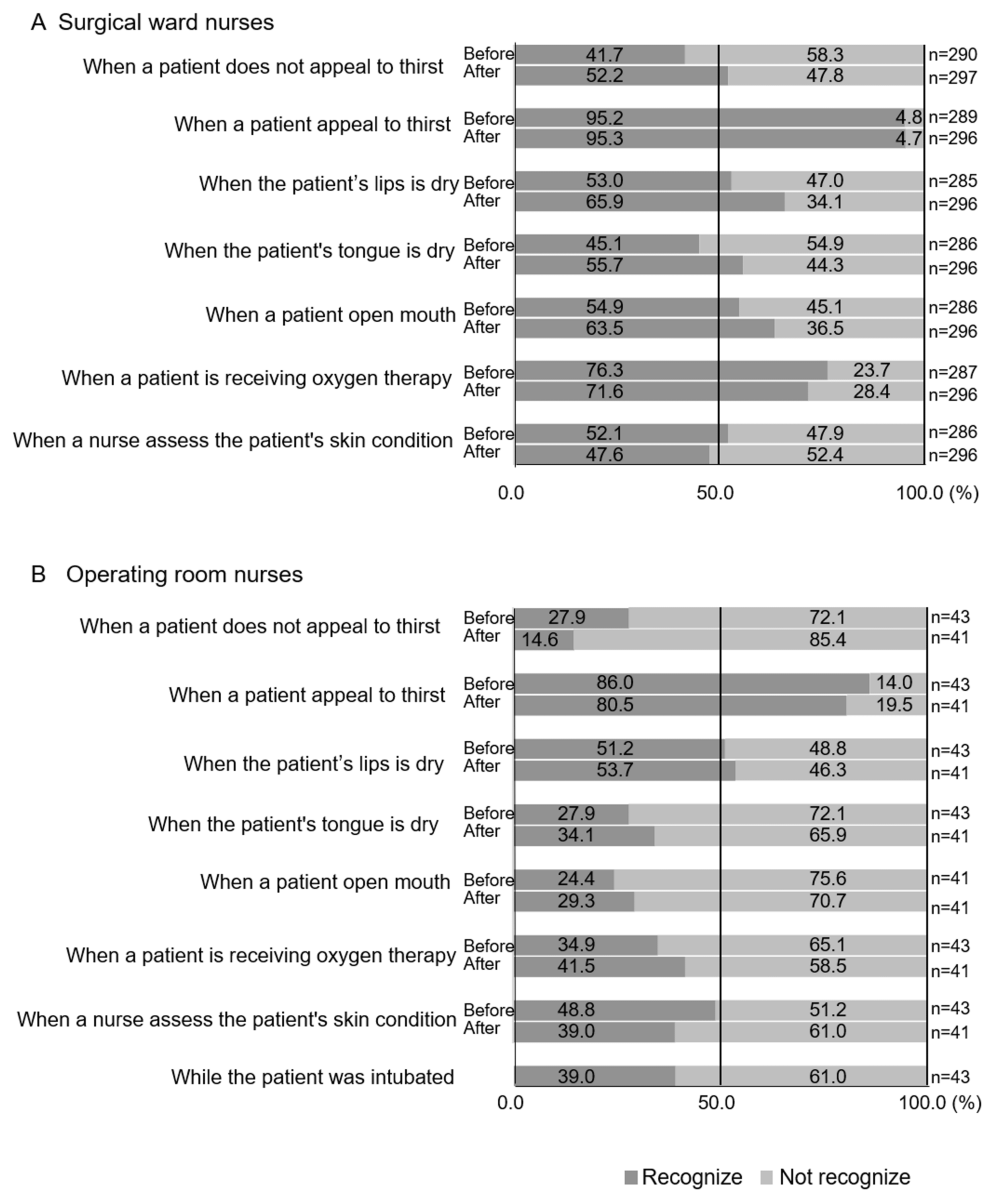
Timing of nurses recognize the patient’s thirst.

Figure 2A is a graph showing the percentage of ward nurses' recognition of patient thirst before and after surgery. Among the ward nurses, the most common timing for recognition of patient thirst before surgery was “When a patient appeals to thirst” (95.2% (275 nurses)), followed by “When a patient is receiving oxygen therapy” (76.3% (219 nurses)). The least common timing was “When a patient does not appeal to thirst” (41.7% (121 nurses)). After surgery, the most common timing for recognition of patient thirst was “When a patient appeals to thirst” (95.3% (282 nurses)), followed by “When a patient is receiving oxygen therapy” (71.6% (212 nurses)). The least common timing was when “When a nurse assesses the patient's skin condition” (47.6% (141 nurses)).

Figure 2B is a graph showing the percentage of operating room nurses' recognition of patient thirst before and after surgery. Among the operating room nurses, the most common timing for recognition of patient thirst upon entering the operating room was “When a patient appeals to thirst” (86.0% (37 nurses)), followed by “When the patient’s lips is dry” (51.2% (22 nurses)). The most common timing for recognition of patient thirst from the end of surgery until the patient’s exit from the operating room was “When a patient appeals to thirst” (80.5% (33 nurses)), followed by “When the patient’s lips is dry” (53.7% (22 nurses)). Furthermore, only 16 nurses (39.0%) recognized patient thirst (oral dryness) during surgery (while the patient was intubated).

Sharing of Nursing Information About Patients’ Thirst

**Figure 3:**
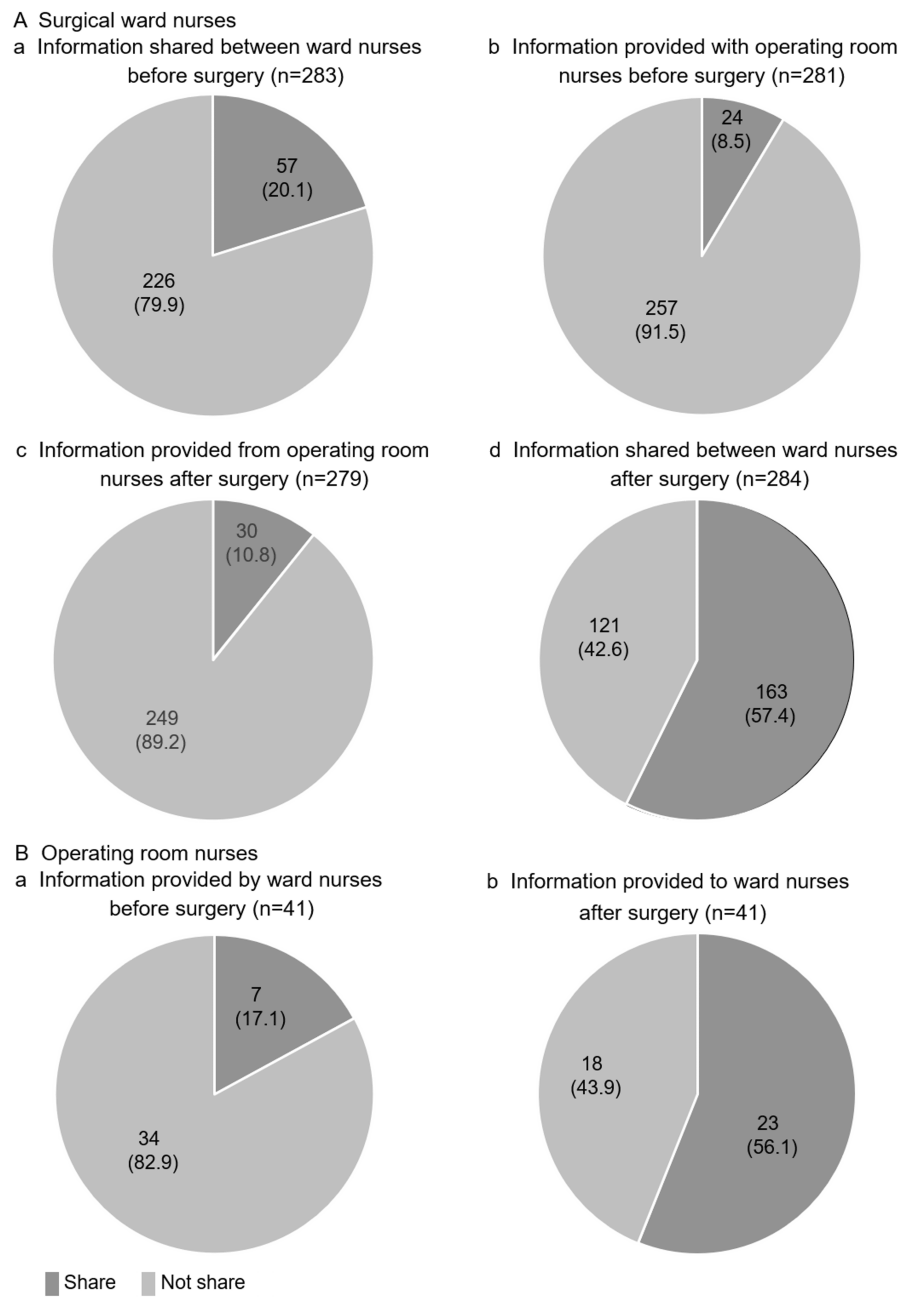
Sharing of nursing information about patients’ thirst, n (%).

Figure 3A shows the information sharing on patient thirst perceived by ward nurses. Regarding the information shared between ward nurses before surgery, the majority of ward nurses (226 nurses (79.9%)) answered that they did not share information about thirst. Information provided with operating room nurses before surgery by ward nurses to operating room nurses, 257 ward nurses (91.5%) indicated that they had never shared this information. Regarding the information provided from operating room nurses to ward nurses after surgery, 249 ward nurses (89.2%) answered that they did not share information. Regarding the information shared between ward nurses after surgery, a high percentage of nurses shared information (163 nurses (57.4%)).

Figure 3B shows the information sharing on patient thirst perceived by operating room nurses. Regarding the information provided by ward nurses before surgery, many operating room nurses answered that they did not share the information (34 nurses (82.9%)). On the other hand, 23 operating room nurses responded that they share information about thirst with ward nurses after surgery (56.1%).

Evaluation of Patient Thirst 

To determine whether scales or scores were used to evaluate patient thirst during information sharing, the participants were asked whether they had ever used a scale or score to evaluate thirst in their work. None of the participants reported having used a scale or score to evaluate thirst.

Care for thirst in patients during the perioperative period

Questions were posed regarding the nursing care provided for patients' thirst during the perioperative period and the reasons behind the care actually administered by nurses.

Actual Care Provided to Patients for Preoperative Thirst

Table 2A shows the nurses' actual care for the patient's preoperative thirst and the reasons for it. There were 114 ward nurses who provided care for patients with thirst before surgery. The most common type of care provided was “mouthwash with water,” which was reported by 111 nurses (97.4%). The reasons for choosing this type of care were “because of the patient's wishes” by 81 nurses (71.1%) and “because I think it is effective” by 69 nurses (60.5%). There were only five operating room nurses who provided care for patients with thirst before surgery. The most common type of care provided was “placing moist gauze against the mouth” (four nurses (80.0%)). The main reason for choosing this type of care was “because I think it is effective” (three nurses (60.0%)). There were 169 ward nurses who did not provide care for patients with thirst before surgery. The most common reason for not providing care was “because there is an instruction not to drink water” (54 nurses (32.7%)). There were 35 operating room nurses who did not provide thirst care to preoperative patients. The reasons for not providing care were “because there is an instruction not to drink water” (10 nurses (38.5%)) and “I don't have the knowledge and don't know how” (six nurses (23.1%)).

**Table 2:** Care provided to patients with thirsty before surgery. The data are presented as n (%). †: This option is a selectable item.

A. Nurses who provided care	Surgical ward nurse (n=114)	Operating room nurse (n=5)
	n (%)	n (%)
Type of care^†^		
Placed a moistened gauze against the mouth	20 (17.5)	4 (80.0)
Performed oral cleaning using a wet sponge brush	14 (12.3)	0
They were asked to do a mouthwash with water	111 (97.4)	0
They were asked to do a mouthwash with mouthwash	8 (7.0)	0
Used a mouth moisturizing spray	3 (2.6)	0
Applying a protective agent (Vaseline, etc.) to the lips	16 (14.0)	1 (20.0)
Others	6 (5.3)	1 (20.0)
Reasons for choosing care^†^		
Because of the patient's wishes	81 (71.1)	2 (40.0)
Because I was taught in school when I was a student	1 (0.9)	0
Because I learned it from my senior	29 (25.4)	1 (20.0)
Because it's a rule on the ward	6 (5.3)	0
Because I have seen it in literature or other sources	3 (2.6)	0
Because I think it is effective	69 (60.5)	3 (60.0)
Somehow	6 (5.3)	0
Others	3 (2.6)	1 (20.0)
B. Nurses who did not provide care	Surgical ward nurse (n=169)	Operating room nurse (n=35)
	n (%)	n (%)
Reasons for not taking care^†^		
Because there is an instruction not to drink water	54 (32.7)	10 (38.5)
Because of hospital rules	0	0
Because I was taught not to care	0	0
Somehow	6 (3.6)	2 (7.7)
I don't have the knowledge and don't know how	13 (7.9)	6 (23.1)
Because you don't think it's necessary	7 (4.2)	2 (7.7)
Due to the possibility of aspiration	5 (3.0)	3 (11.5)
Because you think it won't work even if you go	0	1 (3.8)
Because I'm busy with other work	7 (4.2)	3 (11.5)
Others	19 (11.5)	6 (23.1)
No answer	4 (2.4)	9 (34.6)

*Actual Care Provided to Patients With Thirst During Surgery* 

Fourteen of the operating room nurses never or rarely provided care. Only six nurses reported that they provided care during surgery by "placed a moistened gauze against the mouth'' (four nurses (66.7%)) and "applying a protective agent (Vaseline, etc.) to the lips'' (three nurses (50.0%)). The reason for choosing to provide care was "because I think it is effective'' (four nurses (66.7%)).

Actual Care Provided to Patients for Postoperative Thirst

Table 3A shows the nurses' actual care for the patient's postoperative thirst and the reasons for it. There were 200 ward nurses who responded regarding the actual care provided to patients for postoperative thirst. The most common type of care provided was “mouthwash with water,” which was administered to 189 nurses (94.5%). The reasons for choosing this type of care were “patient’s request” for 165 nurses (82.5%), “thought to be effective” for 97 nurses (48.5%), and “taught by senior staff” for 55 nurses (27.5%). There were eight operating room nurses who responded regarding the actual care provided to patients for postoperative thirst. The most common type of care provided was “placing moist gauze against the mouth,” which was administered to seven nurses (87.5%). The reasons for choosing this type of care were “thought to be effective” for three nurses (37.5%), “taught by senior staff” for two nurses (25.0%), and “ward’s rule” for two nurses (25.0%).

Table 3B shows the reasons why nurses did not provide care for patients' preoperative thirst. There were 82 ward nurses who did not care provided to patients for postoperative thirst. The reasons for not providing care were “prohibition of water intake” for 39 nurses (47.6%), followed by “risk of aspiration” for 20 nurses (24.4%). There were 35 operating room nurses who did not care provided to patients for postoperative thirst. The reasons for not providing care were “prohibition of water intake” for 10 nurses (28.6%), “risk of aspiration” for six individuals (17.1%), and “not knowing how to provide care” for five nurses (14.3%).

**Table 3:** Care provided to patients with thirsty after surgery. The data are presented as n (%). †: This option is a selectable item; ‡: This is an option that cannot be answered.

A. Nurses who provided care	Surgical ward nurse (n=200)	Operating room nurse (n=8)
	n (%)	n (%)
Type of care^†^		
Placed a moistened gauze against the mouth	80 (40.0)	7 (87.5)
Performed oral cleaning using a wet sponge brush	51 (25.5)	0
They were asked to do a mouthwash with water	189 (94.5)	0
They were asked to do a mouthwash with mouthwash	9 (4.5)	0
Using an oral moistening spray	3 (1.5)	0
Applying a protective agent (Vaseline, etc.) to the lips	25 (12.5)	2 (25.0)
Sprayed saline into the oral cavity	2 (1.0)	0
Spray lemon water into the mouth	0 (0.0)	0
I asked my doctor if I could drink water	39 (19.5)	2 (25.0)
Others	6 (3.0)	1 (12.5)
Reasons for choosing care^†^		
Because of the patient's wishes	165 (82.5)	^‡^
Because I was taught in school when I was a student	3 (1.5)	1 (12.5)
Because I learned it from my senior	55 (27.5)	2 (25.0)
Because it's a rule on the ward	5 (2.5)	2 (25.0)
Because I have seen it in literature or other sources	4 (2.0)	1 (12.5)
Because I think it is effective	97 (48.5)	3 (37.5)
Somehow	10 (5.0)	1 (12.5)
Others	4 (2.0)	2 (25.0)
B. Nurses who did not provide care	Surgical ward nurse (n=82)	Operating room nurse (n=35)
	n (%)	n (%)
Reasons for not taking care^†^		
Because there is an instruction not to drink water	39 (47.6)	10 (28.6)
Because of hospital rules	1 (1.2)	0 (0.0)
Because I was taught not to care	0 (0.0)	0 (0.0)
Somehow	3 (3.7)	2 (5.7)
I don't have the knowledge and don't know how	7 (8.5)	5 (14.3)
Because you don't think it's necessary	4 (4.9)	2 (5.7)
Due to the possibility of aspiration	20 (24.4)	6 (17.1)
Because you think it won't work even if you go	0 (0.0)	2 (5.7)
Because I'm busy with other work	9 (11.0)	3 (8.6)
Others	7 (8.5)	5 (14.3)

## Discussion

In this study, we aimed to clarify nurses’ recognition and care practices for patient thirst during the perioperative period, focusing on nurses caring for perioperative patients. The participants in this study were experienced nurses with an average of 10-12 years of nursing experience, regardless of their affiliation. Most of the nurses from all affiliations had graduated from university. Therefore, the results of this study can be considered as insights from experienced nurses.

Nurses mainly observed “postoperative pain,” “nausea and vomiting,” “agitation and delirium,” and “bleeding” as postoperative observation items, while “thirst” was observed less frequently than the other observation items. This result is similar to the finding that nurses underestimate patient thirst [9]. In addition, nurses frequently observed items with clear preventive measures and treatment plans. For example, the management of “agitation/delirium” was added to clinical guidelines in 2015 by the Japanese Society of Intensive Care Medicine. "Postoperative pain" was added as an additional management item under medical fees, and ondansetron was covered for postoperative "nausea and vomiting" in Japan [26] in 2021. Therefore, these observation items are likely to be observed more frequently by the nurse. These results indicate that nurses observe items with clearly defined preventive measures and treatment strategies more frequently. However, evidence for the care of “thirst” is not organized [27], and no clear method of improvement has been shown. In addition, the Perioperative Management Team Textbook (4th edition, 2020) clearly states that “thirst” occurs, but there is no clear method of improvement for thirst described in the text. Therefore, it was inferred that patients' thirst was infrequently observed postoperatively and was not considered as an item to be evaluated. From the results of this study, it became evident that 24 of 43 operating room nurses (55.8%) were “rarely observed” and six (14.0%) “never observed”. For operating room nurses, the situation in which a patient is intubated is routine and is not physically complained of by the patient during anesthesia. Therefore, it is inferred that operating room nurses place a lower priority on observing thirst than ward nurses, resulting in lower awareness and less frequent observation.

Both ward nurses and operating room nurses had a higher recognition rate of patient thirst when the patients reported it. Additionally, recognition rates based on physical observations, such as dry lips or tongue and skin conditions, are lower than the rate of recognition based on patient complaints. In particular, operating room nurses had a recognition rate of less than 50% for recognizing thirst based on physical observations, including tongue and skin condition and the patient’s mouth being open. Operating room nurses are expected to respond quickly to life-threatening emergencies, such as cardiovascular or respiratory changes [28]. Therefore, it is assumed that operating room nurses have low awareness of thirst in non-life-threatening patients. Previous studies have indicated that anesthesiologists and ward nurses recognize patient thirst only when it is reported by the patients, as seen in the present study. Additionally, ward nurses had a recognition rate of 70% or higher for the item “when the patient is undergoing oxygen therapy,” suggesting that they may have associated the patient’s state of receiving oxygen therapy with experiencing thirst. Negro et al. demonstrated a correlation between the use of humidified Venturi masks and thirst, but they found no association with NIV or Venturi masks [29]. Based on this prior research, it is believed that there may be a discrepancy between patients’ complaints and nurses’ recognition of thirst, emphasizing the importance of intentional listening to facilitate the reporting of thirst by patients.

The sharing of nursing information regarding patients' thirst was perceived differently between ward nurses and operating room nurses. Information sharing regarding patients' thirst between different departments, wards, and operating rooms was low. About 89.2% of ward nurses responded that they did not receive information about patients' thirst from operating room nurses, while 56.1% of operating room nurses responded that they shared information, revealing a difference in perception between the two groups of nurses. Information sharing regarding patients' thirst within the ward may be shared among ward nurses, as well as postoperative status and monitoring. Nurses stated that "there is a dilemma in which information cannot be shared due to differences in risk perception between the operating room and the ward [28]." Therefore, there may be a discrepancy between what operating room nurses think they are sharing with ward nurses and what ward nurses think they are receiving.

All nurses evaluated thirst without using any scales or measures. This result indicates that Japanese nurses are not in customs of using a scale to assess patients' thirst. Thirst is a subjective experience influenced by individual health status and physiological, psychological, social, and cultural factors [30]. More than 70% of the nurses had no experience with general anesthesia (Table 1). Therefore, although nurses were able to recognize the presence of thirst from patients' complaints, it was difficult to imagine the level of discomfort or assess the severity of thirst. Previous studies have used numerical rating scales (NRS) and visual analog scales (VAS) to assess thirst and pain and have shown their usefulness [14]. However, the frequency of observations of thirst was low (Figure 1). This result suggests that objective rating scales might not be used daily to assess patient's thirst after surgery in clinical settings.

Regarding the actual state of perioperative care for patients' thirst by nurse type, in this survey, many nurses did not care for patients' thirst. The most common reason for not providing preoperative care was "because I was instructed not to drink fluids." It was suggested that nurses did not care for patients' thirst in accordance with the doctor's instructions for patient safety, as absolute fluid intake restrictions are recommended for preoperative patients from two hours before surgery. After surgery, 67.1% (200 of 298) of the surgical ward nurses and 18.6% (eight of 43) of the operating room nurses provided care for patient thirst. The Perioperative Management Team Textbook 4th edition (Japanese Society of Anesthesiologists, 2020) states that the strict dietary restrictions imposed on patients before surgery and anesthesia are gradually lifted according to the patient’s recovery after surgery. Therefore, nurses are responsible for considering the timing of initiating oral intake for patients and consulting with physicians. In this study, the proportion of nurses who provided care after surgery was higher than before surgery, and it was speculated that the nurses provided care based on their judgment. The reasons for choosing care were “patient’s wishes” and “because they believed it was effective,” which were common among ward nurses and operating room nurses. Pavani et al.’s study (2016) [9] showed that nurses empirically provide care for patient thirst. The study participants were experienced nurses, and it was inferred that they provided care for patient thirst based on their experience.

Conversely, the reason for not providing care for postoperative thirst was often due to “instruction not to drink water” and “the possibility of aspiration,” and it was suggested that some nurses prioritize patient safety over providing care. Current anesthesia allows for early awakening, and early recovery programs, such as Early Recovery After Surgery, have been advocated. Nascimento et al. (2014) [15] created a “Safe Protocol for Thirst Management” to confirm the level of consciousness, airway reflex, and nausea/ vomiting immediately after surgery to improve thirst management. Yoshisuke et al. (2020) [19] suggested that thirst was improved by early postoperative drinking and reducing the oxygen inhalation time. It is only recently that evidence-based care and program development to significantly improve thirst have been conducted [14]. This study revealed that nurses provide care for postoperative thirst based on their own experience. Therefore, it is suggested that observing physical signs related to thirst and combining them with nursing experience is necessary to provide evidence-based care in the future.

This study has some limitations that should be noted. First, the study was conducted at a university hospital. Therefore, it is necessary to clarify the actual situation of care for thirst on a broader scale through further investigation. Second, this study targeted nurses, and there is a possibility that their recognition of patient thirst differs from the recognition of the patients themselves. Future studies are needed to assess consistent whether nurses and patients’ recognition for thirst.

## Conclusions

Postoperative thirst is a common and distressing symptom. However, the results of this study revealed that ward nurses and operating room nurses had low awareness of postoperative thirst and mainly relied on patients' verbal complaints rather than active physical observation. Furthermore, there was a gap in the sharing of nursing information between ward nurses and operating room nurses. Due to a lack of knowledge and fear of complications, both types of nurses provided care based on their own experience rather than evidence-based care.

## Appendices

**Table 4:** Self-administered questionnaire.

Questionnaire
Q1. Background of the participants
(Gender, years of nursing experience, last school institution, qualifications other than nurses, and general anesthesia experience)
Q2. What are the postoperative observations in daily practice? (Frequency)
4: Always observe; 3: Sometimes observe; 2: Rarely observe; 1: Never observe
1) Postoperative pain
2) Nausea/Vomiting
3) Thirst
4) Restlessness, delirium
5) Signs of infection
6) Bleeding
Q3. Do you use a scale or scale to assess thirst in your daily work?
1) No scale or scale may be used to evaluate
2) I evaluate using a scale or scales
The following questions were based on the respondent's recollection of the care you engaged in during the past month.
Q4. When does a nurse recognize a patient's thirst? (Before surgery/After surgery)
4: Always recognize; 3: Sometimes recognize; 2: Rarely recognize; 1: Never recognize
1. Surgical ward nurses
a) When a patient does not appeals to thirst
b) When a patient appeal to thirst
c) When the patient’s lips is dry
d) When the patient's tongue is dry
e) When a patient opens mouth
f) When a patient is receiving oxygen therapy
g) When a nurse assess the patient's skin condition
2. Operating room nurses
a) When a patient does not appeal to thirst
b) When a patient appeals to thirst
c) When the patient’s lips is dry
d) When the patient's tongue is dry
e) When a patient opens mouth
f) When a patient is receiving oxygen therapy
g) When a nurse assess the patient's skin condition
h) While the patient was intubated
Q5. When do you share information about a patient's thirst? (Share/Not share)
4: Always share; 3: Sometimes share; 2: Rarely share; 1: Never share
1. Surgical ward nurses
a) Information shared between ward nurses before surgery
b) Information provided with operating room nurses before surgery
c) Information provided from operating room nurses after surgery
d) Information shared between ward nurses after surgery
2. Operating room nurses
a) Information provided by ward nurses before surgery
b) Information provided to ward nurses after surgery
Q6. Do you provide care for thirsty patients before surgery? (Surgical ward nurses/Operating room nurses)
1. What care do you provide?
a) Placed a moistened gauze against the mouth
b) Performed oral cleaning using a wet sponge brush
c) They were asked to do a mouthwash with water
d) They were asked to do a mouthwash with mouthwash
e) Used a mouth moisturizing spray
f) Applying a protective agent (Vaseline, etc.) to the lips
g) Others
2. Why did you choose the care?
a) Because of the patient's wishes
b) Because I was taught in school when I was a student
c) Because I learned it from my senior
d) Because it's a rule on the ward
e) Because I have seen it in literature or other sources
f) Because I think it is effective
g) Somehow
h) Others
3. Why do you not provide the care?
a) Because there is an instruction not to drink water
b) Because of hospital rules
c) Because I was taught not to care
d) Somehow
e) I don't have the knowledge and don't know how
f) Because you don't think it's necessary
g) Due to the possibility of aspiration
h) Because you think it won't work even if you go
i) Because I'm busy with other work
j) Others
k) No answer
Q7. Care provided to patients with thirsty after surgery (Surgical ward nurses/Operating room nurses)
1. What care do you provide?
a) Placed a moistened gauze against the mouth
b) Performed oral cleaning using a wet sponge brush
c) They were asked to do a mouthwash with water
d) They were asked to do a mouthwash with mouthwash
e) Using an oral moistening spray
f) Applying a protective agent (Vaseline, etc.) to the lips
g) Sprayed saline into the oral cavity
h) Spray lemon water into the mouth
i) I asked my doctor if I could drink water
j) Others
2. Why did you choose the care?
a) Because of the patient's wishes
b) Because I was taught in school when I was a student
c) Because I learned it from my senior
d) Because it's a rule on the ward
e) Because I have seen it in literature or other sources
f) Because I think it is effective
g) Somehow
h) Others
3. Why do you not provide the care?
a) Because there is an instruction not to drink water
b) Because of hospital rules
c) Because I was taught not to care
d) Somehow
e) I don't have the knowledge and don't know how
f) Because you don't think it's necessary
g) Due to the possibility of aspiration
h) Because you think it won't work even if you go
i) Because I'm busy with other work
j) Others

**Table 5:** STROBE statement—checklist of items that should be included in reports of case-control studies. *Give information separately for cases and controls. STROBE: Strengthening the Reporting of Observational Studies in Epidemiology. Note: An explanation and elaboration article discusses each checklist item and gives methodological background and published examples of transparent reporting. The STROBE checklist is best used in conjunction with this article (freely available on the Web sites of PLoS Medicine at http://www.plosmedicine.org/, Annals of Internal Medicine at http://www.annals.org/, and Epidemiology at http://www.epidem.com/). Information on the STROBE Initiative is available at http://www.strobe-statement.org.

Items	Recommendation	Page
1. Title and abstract	(a) Indicate the study’s design with a commonly used term in the title or the abstract	Title
	(b) Provide in the abstract an informative and balanced summary of what was done and what was found	Abstract
Introduction		
2. Background/rationale	Explain the scientific background and rationale for the investigation being reported	Introduction
3. Objectives	State-specific objectives, including any prespecified hypotheses	Introduction
Methods		
4. Study design	Present key elements of study design early in the paper	Method
5. Setting	Describe the setting, locations, and relevant dates, including periods of recruitment, exposure, follow-up, and data collection	Method
6. Participants	(a) Give the eligibility criteria, and the sources and methods of case ascertainment and control selection. Give the rationale for the choice of cases and controls	Method
	(b) For matched studies, give matching criteria and the number of controls per case	None
7. Variables	Clearly define all outcomes, exposures, predictors, potential confounders, and effect modifiers. Give diagnostic criteria, if applicable	Method
8. Data sources/measurement*	For each variable of interest, give sources of data and details of methods of assessment (measurement). Describe comparability of assessment methods if there is more than one group	Method
9. Bias	Describe any efforts to address potential sources of bias	Method
10. Study size	Explain how the study size was arrived at	Method
11. Quantitative variables	Explain how quantitative variables were handled in the analyses. If applicable, describe which groupings were chosen and why	Method
12. Statistical methods	(a) Describe all statistical methods, including those used to control for confounding	Method
	(b) Describe any methods used to examine subgroups and interactions	Method
	(c) Explain how missing data were addressed	Method
	(d) If applicable, explain how the matching of cases and controls was addressed	Method
	(e) Describe any sensitivity analyses	Method
Results		
13. Participants*	(a) Report numbers of individuals at each stage of study—e.g., numbers potentially eligible, examined for eligibility, confirmed eligible, included in the study, completing follow-up, and analyzed	Result
	(b) Give reasons for non-participation at each stage	Result
	(c) Consider the use of a flow diagram	Result
14. Descriptive data*	(a) Give characteristics of study participants (e.g., demographic, clinical, social) and information on exposures and potential confounders	Result
	(b) Indicate the number of participants with missing data for each variable of interest	Result
15. Outcome data*	Report numbers in each exposure category or summary measures of exposure	None
16. Main results	(a) Give unadjusted estimates and, if applicable, confounder-adjusted estimates and their precision (e.g., 95% confidence interval). Make clear which confounders were adjusted for and why they were included.	None
	(b) Report category boundaries when continuous variables were categorized.	Result
	(c) If relevant, consider translating estimates of relative risk into absolute risk for a meaningful time period.	None
17. Other analyses	Report other analyses done—e.g., analyses of subgroups and interactions and sensitivity analyses	Result
Discussion		
18. Key results	Summarise key results with reference to study objectives	Discussion
19. Limitations	Discuss the limitations of the study, taking into account sources of potential bias or imprecision. Discuss both the direction and magnitude of any potential bias	Discussion
20. Interpretation	Give a cautious overall interpretation of results considering objectives, limitations, multiplicity of analyses, results from similar studies, and other relevant evidence	Discussion
21. Generalisability	Discuss the generalisability (external validity) of the study results	Discussion
Other information		
22. Funding	Give the source of funding and the role of the funders for the present study and, if applicable, for the original study on which the present article is based	Payments
